# Importance of amino acids in brain parenchyma invasion by cancer cells

**DOI:** 10.18632/oncoscience.530

**Published:** 2021-03-31

**Authors:** Alessio Paone, Amani Bouzidi, Serena Rinaldo, Giorgio Giardina, Francesca Cutruzzolà

**Affiliations:** ^1^Department of Biochemical Sciences, A. Rossi Fanelli Laboratory Affiliated to Istituto Pasteur Italia, Sapienza University of Rome, Rome 00185, Italy

**Keywords:** amino acids, brain metastasis, cell migration, chemo-kinesis, brain to blood efflux

Metastases are the scariest manifestation of malignant tumors and in particular those developing in the brain are critical for patient’s survival, due to the delicacy of the organ and its connection to vital functions, which make therapeutic and surgery intervention particularly difficult.

Metastasis formation occurs in a multi-step process. The cells leave the primary tumor entering the bloodstream and are dispersed throughout the body, then extravasate invading the target organ and finally grow developing the actual metastasis. Specific tumor types such as lung, breast and melanoma have a propensity to form brain metastases even if the mechanism that determines this specific tropism has not yet been clarified. The cerebral microenvironment seems to play a fundamental role, as cancer cells are able to adapt their metabolism to exploit the available resources. The brain extracellular fluid is enriched in specific molecules such as amino acids and neurotransmitters which, together with other molecules, can be employed by tumor cells to support fundamental catabolic and anabolic reactions.

**Figure 1:**
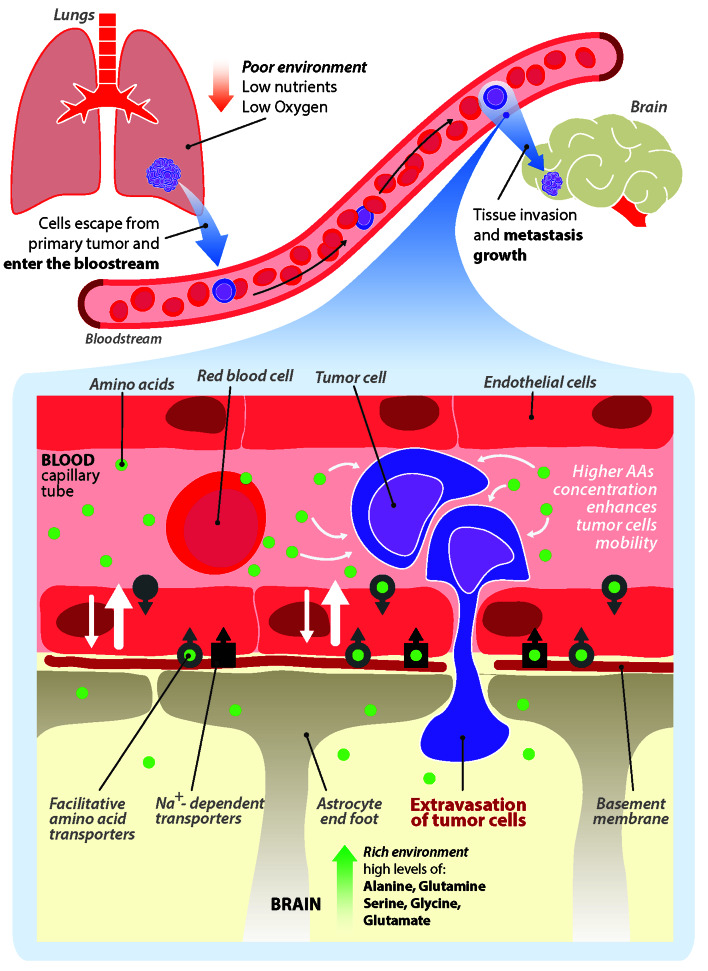
(modified from [3]) Schematic representation of the brain to blood efflux- metastatic hypothesis. chemo-kinetic ability of metastatic cancer cells is increased in the brain microvasculature in presence of increased amount of amino acids triggering the extravasation process

Extracellular amino acids are recently emerging as key players not only in supporting the growth of metastatic cells in the brain parenchyma, but also in the process of extravasation and invasion. The first amino acids identified to be involved in this aspect were asparagine and glutamate [1, 2]. We have recently shown that extracellular serine and glycine are able to stimulate the chemo-kinesis of lung cancer cells through a mechanism involving Serine hydroxymethyltransferase isoform 1 (SHMT1), the protein responsible for serine/glycine interconversion in the cytoplasm [3]. Cytoplasmic serine availability controls the migratory ability of lung cancer cells increasing ATP production and reducing ROS formation and we hypothesize that this process is closely linked to the extravasation process and involved in the selection of the target organ. Our hypothesis is based on the concept of brain to blood efflux (BBE) (Figure 1). In the brain, the level of amino acids in the extracellular fluid must be kept low to avoid unwanted stimuli or the activation of toxic mechanisms for brain cells [4]. Neurotransmitters and amino acids are rapidly re-uptaken by the cells of the microenvironment through specific transporters. The endothelial cells in the brain display at the abluminal side the same transporters expressed on brain cells and participate in the BBE mechanism, by absorbing the excess of molecules from the brain extracellular fluid and releasing it into the bloodstream [4]. The sodium dependent transporters of the ASC family (SLC1A4 and SLC1A5), for example, are responsible for alanine, serine and cysteine efflux, while the excitatory amino acid transporter (EAAT) family is responsible of glutamate efflux and the sodium neutral amino acid transporter 3 (SNAT3) is involved in alanine, proline, histidine, serine, asparagine efflux [5, 6]. It is known that cancer cells are able to reach the microvasculature of the brain and adhere to it, often forming micro clots [7]. We hypothesize that a local decrease of the blood flow due to a micro clot, together with the continuous release of amino acids through the BBE mechanism, leads to a local increase in the concentration of amino acids in the microvasculature; this enrichment may be sufficient to provide the metabolites required to initiate the invasion process by dramatically increasing the chemo-kinetic ability of cancer cells.

Our data clearly demonstrated that also a modest decrease in cytoplasmic serine content can induce the activation of AMP kinase that completely inhibits the migratory process [3]. From the clinical point of view, blocking the extravasation process through inhibitor(s) of amino acid importers would help limiting metastasis formation for example in those patients awaiting the surgical resection of the primary tumor. Considering the incidence of the metastatic phenomenon, this proof of concept could have a substantial impact on the survival of patients affected by melanoma, lung or breast cancer of which are currently the most widespread and with the highest mortality rate.
